# Efficacy and safety of chronomodulated irinotecan, oxaliplatin, 5‐fluorouracil and leucovorin combination as first‐ or second‐line treatment against metastatic colorectal cancer: Results from the International EORTC 05011 Trial

**DOI:** 10.1002/ijc.33422

**Published:** 2020-12-10

**Authors:** Pasquale F. Innominato, Abdoulaye Karaboué, Christian Focan, Philippe Chollet, Sylvie Giacchetti, Mohamed Bouchahda, Ayhan Ulusakarya, Angela Torsello, René Adam, Francis A. Lévi, Carlo Garufi

**Affiliations:** ^1^ North Wales Cancer Centre, Ysbyty Gwynedd, Betsi Cadwaladr University Health Board Bangor UK; ^2^ Cancer Chronotherapy Team, Cancer Research Centre, Division of Biomedical Sciences Warwick Medical School Coventry UK; ^3^ UPR “Chronotherapy, Cancers and Transplantation”, Faculty of Medicine Paris‐Saclay University Villejuif France; ^4^ Medical Oncology Unit GHI Le Raincy‐Montfermeil Montfermeil France; ^5^ Department of Oncology CHC‐MontLegia, Groupe Santé CHC‐Liège Liège Belgium; ^6^ Clinical and Translational Research Division Jean Perrin Comprehensive Cancer Centre Clermont‐Ferrand France; ^7^ Department of Oncology Saint Louis Hospital, Public Hospitals of Paris (AP‐HP) Paris France; ^8^ Medical Oncology Unit Clinique du Mousseau Evry France; ^9^ Medical Oncology Unit Clinique Saint Jean L'Ermitage Melun France; ^10^ Chronotherapy Unit, Department of Medical Oncology Paul Brousse Hospital, Public Hospitals of Paris (AP‐HP) Villejuif France; ^11^ Division of Medical Oncology San Giovanni‐ Addolorata Hospital Rome Italy; ^12^ Hepatobiliary Centre Paul Brousse Hospital, Public Hospitals of Paris (AP‐HP) Villejuif France; ^13^ Division of Medical Oncology San Camillo Forlanini Hospital Rome Italy

**Keywords:** chemotherapy, chronotherapy, circadian, colorectal cancer, FOLFIRINOX

## Abstract

The triplet combination of irinotecan, oxaliplatin and fluorouracil is an active frontline regimen in metastatic colorectal cancer, but scarce data exist on its use as salvage treatment. We aimed at assessing its safety and efficacy profiles with its circadian‐based administration (chronoIFLO5) as either first‐ or second‐line treatment, within the time‐finding EORTC 05011 trial. Five‐day chronoIFLO5 was administered every 3 weeks in patients with PS 0, 1 or 2. It consisted of chronomodulated irinotecan (180 mg/sqm), oxaliplatin (80 mg/sqm) and fluorouracil‐leucovorin (2800 and 1200 mg/sqm, respectively). For our study, toxicity and antitumour activity were evaluated separately in first‐ and second‐line settings. Primary endpoints included Grade 3‐4 toxicity rates, best objective response rate (ORR), progression‐free survival (PFS) and overall survival (OS). One‐hundred forty‐nine and 44 patients were treated in first‐line and second‐line settings, respectively, with a total of 1138 cycles with median relative dose intensities of about 90%. Demographics were comparable in the two groups. Thirty‐six (24.7%) and 10 (22.2%) patients experienced at least one episode of severe toxicity in first line and second line, respectively. Frontline chronoIFLO5 yielded an ORR of 62.3% [95% CI: 54.2‐70.4] and resulted in median PFS and OS of 8.7 months [7.5‐9.9] and 19.9 months [15.4‐24.5]. Corresponding figures in second line were 37.5% [22.5‐52.5], 6.7 months [4.8‐8.9] and 16.3 months [11.8‐20.8]. International and prospective evaluation revealed the favourable safety and efficacy profiles of chronoIFLO5, both as frontline and as salvage treatment against metastatic colorectal cancer. In particular, encouraging activity in second line was observed, with limited haematological toxicity.

## Abstract 2

**What's new?**

Triple chemotherapy of irinotecan, oxaliplatin and fluorouracil‐leucovorin achieves the best survival against metastatic colorectal cancer (CRC) but has worse toxicity than doublet therapy. Chronomodulation is a strategy to reduced toxicity by coordinating drug administration with the patient's circadian rhythm. Here, the authors evaluated the safety and efficacy of chronomodulated triple therapy given over a 5‐day period every 3 weeks. 149 patients received the treatment as a first‐line regimen, while 44 received it as salvage therapy. In both settings, the safety and efficacy of chronomodulated triple therapy were validated, although the optimal timing of irinotecan for minimizing toxicity remains to be determined.

**Abbreviations**
chronoIFLO5: chronomodulated irinotecan, fluorouracil, leucovorin, oxaliplatin over 5 days
EGFR: epithelial growth factor receptor
EORTC: European Organisation for Research and Treatment of Cancer
FOLFIRINOX: irinotecan, fluorouracil, leucovorin, oxaliplatin
FOLFOXIRI: folinic acid, 5‐fluorouracil, oxaliplatin and irinotecan
G‐CSF: granulocyte colony‐stimulating factor
GI: gastrointestinal
NCI CTC AE: National Cancer Institute Common Terminology Criteria for Adverse Events
ORR: objective response rate
OS: overall survival
PFS: progression‐free survival
PS: performance status
RECIST: Response Evaluation Criteria in Solid Tumours
sqm: square metre
VEGF: vascular endothelial growth factor

## 1 BACKGROUND

Colorectal cancer is the second most common neoplastic disease across Europe in terms of both incidence and mortality.^1^ Systemic chemotherapy is the principal therapeutic option for metastatic disease, and it is best tailored to patient's and disease's features within a multidisciplinary oncosurgical strategy.^2^ One such approach is to intensify chemotherapy with the aim of obtaining substantial downsizing to allow conversion from nonresectable to resectable disease.^3^ The combination of the three main cytotoxic drugs active against colorectal cancer, 5‐fluorouracil, oxaliplatin and irinotecan, constitutes the chemotherapy backbone, which has achieved the best outcomes in the metastatic setting.^4^, ^5^ Nonetheless, the triplet regimen has also been associated with worse toxicity in comparison with doublets.^6^ Hence, better patient outcomes could be expected with the improved tolerability of the triplet regimen.

The administration of each chemotherapy drug at a defined time based on its circadian tolerability constitutes the rationale of chronotherapy.^7^, ^8^, ^9^ Indeed, the chronomodulated triplet has demonstrated satisfactory safety and efficacy in monocentric studies in patients with metastatic colorectal cancer.^10^, ^11^, ^12^, ^13^ Based on this evidence, we conducted an international time‐finding study (EORTC 05011) to identify the least toxic administration time of Irinotecan, combined with chronomodulated oxaliplatin and 5‐fluorouracil + leucovorin.^14^ This trial failed to meet its primary endpoint of determining the time of irinotecan delivery causing the lowest toxicity in the whole population.^14^ Nevertheless, in accordance with recent evidence of the impact of gender in outcomes of chemotherapy against colorectal cancer, we found a lag in the least toxic time of irinotecan administration according to gender. Thus, lower toxicity of irinotecan was highlighted following dosing in the early morning for men and in the afternoon for women.^14^

Here, we performed a final update of the EORTC 05011 trial data, which complements overall safety and efficacy of chronomodulated triplet both in the first‐line setting, comparatively to existing data with conventional administration, and as a second‐line regimen, for which scant prospective multicentric data exist.

## 2 METHODS

### 2.1 Study population

**FIGURE 1:**
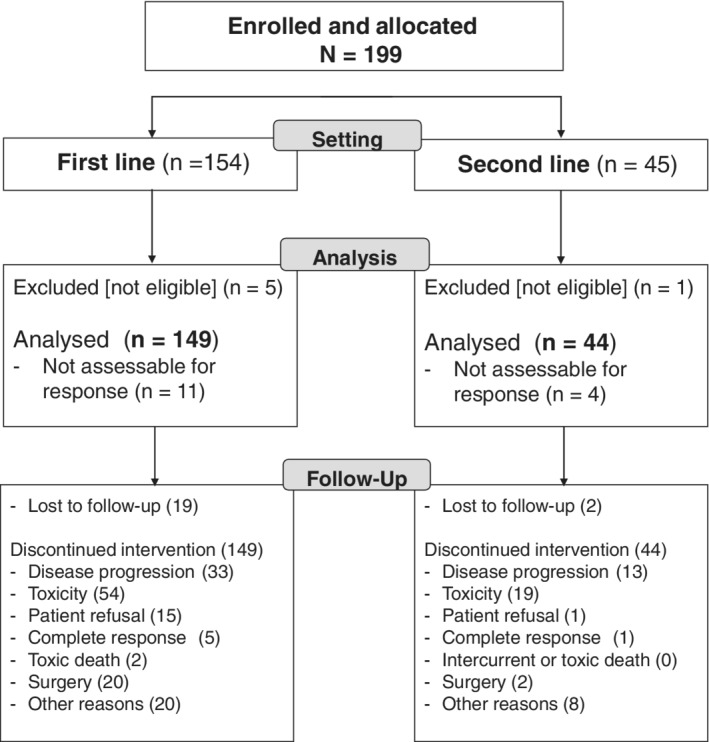
CONSORT diagram

The EORTC 05011 trial involved 18 institutions in Europe, which enrolled a total of 199 adult patients with histologically proven, measurable and unresectable advanced colorectal cancer and good (ie, <3 on the World Health Organisation scale) performance status, between February 2002 and August 2005. Patients could have received up to one prior line of chemotherapy for metastatic disease or locoregional recurrence, including irinotecan‐ or oxaliplatin‐based combination protocols. Adjuvant chemotherapy was considered as first line, if relapse occurred within 6 months of its completion. Patients were required to have adequate hepatic, renal and haematological functions, no baseline diarrhoea >Grade 1 (NCIC CTCAE v2) and no prior toxicity related to irinotecan ≥Grade 3. Patients with uncontrolled medical conditions or psychosocial issues representing a potential risk for study compliance and for patient's safety were excluded. The main endpoint of the study was to identify the least toxic time of administration of irinotecan, and patients were randomised to one of six possible times; details on the sample size calculation and allocation to the six treatment arms are provided elsewhere.^14^ Specifically, 193 patients out of the 199 randomised ones (97%) were considered eligible for tolerability and efficacy evaluation: 149 patients were treated in the first‐line setting and 44 in the second‐line one (Figure 1). The trial was approved by the respective ethics review boards at each centre and/or country. It was conducted in accordance with the Declaration of Helsinki guidelines for experimentation on humans.^15^ Signed informed consent was obtained from every participating patient.

### 2.2 Chemotherapy schedule

**FIGURE 2:**
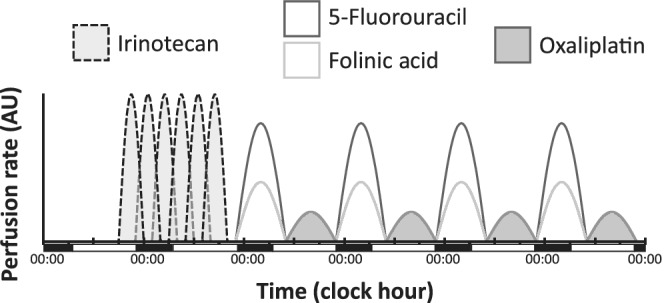
Representation of the chronoIFLO5 protocol. Irinotecan was administered at only one of the six time points in each patient

Chrono‐IFLO5 consisted of the association of irinotecan, administered as a 6‐hour chronomodulated infusion with peak delivery times scheduled every 4 hours according to the allocated treatment arm, followed by the chronomodulated combination of fractionated and alternating 5‐fluorouracil‐leucovorin, peaking at 04:00 am at night, and oxaliplatin, peaking at 4:00 pm, each over 11.5 hours per day for 4 consecutive days (Figure 2). Respective starting doses were 180 mg/sqm for irinotecan, 700 mg/sqm/day for 5‐fluorouracil, 300 mg/sqm/day for leucovorin and 20 mg/sqm/day for oxaliplatin.^14^ The treatments were administered on a full outpatient basis through an ambulatory infusion pump allowing in time programming and delivery of all the medications while the patient was at her/his home (Melodie, Aguettant, France).^16^ Treatment courses were repeated every 3 weeks, that is, after a 16‐day chemotherapy‐free interval. No primary or secondary G‐CSF prophylaxis was allowed.

### 2.3 Outcomes

Efficacy outcome measures included objective response rate, which was calculated using the RECIST v1.1 criteria,^14^ and time‐to‐event endpoints, which comprised progression‐free survival and overall survival. Clinical, haematological and biochemical toxicity was graded before each chemotherapy cycle according to the NCI CTC AE v2 criteria.^14^ Relative dose intensity was calculated as the ratio between the actual dose (expressed in mg/sqm/week) of the three cytotoxic drugs delivered over the whole treatment duration and the theoretical full dose (60 mg/sqm/week for irinotecan, 26.7 mg/sqm/week for oxaliplatin and 933.3 mg/sqm/week for fluorouracil).^14^

### 2.4 Statistical considerations

Efficacy and tolerability outcomes were evaluated separately in the subgroups having received chronoIFLO5 as first‐ and second‐line chemotherapy. Descriptive only statistics were used to characterise the outcomes, and no comparative analysis was performed, given the exploratory nature of this report. Time‐to‐event endpoints were calculated from the day of randomisation up to that of progression (for progression‐free survival) or death (for overall survival). The most recent date with valid follow‐up data was used to censor nonprogressing or alive patients, respectively. The database was frozen in May 2017.

## 3 RESULTS

### 3.1 Study population

Table 1 describes the main clinical and demographic features of the 193 eligible patients, separately in the 149 patients treated in the first‐line setting (77.2%) and in the 44 patients having received the chonomodulated triplet as a second‐line treatment (22.8%) (Table 1). The main reasons for discontinuing chronoIFLO5 included progressive disease (N = 47; 24.4%), severe toxicity (N = 76; 39.3%), including two toxic deaths (1.0%), patient refusal (N = 17; 8.8%) and surgery of metastases (N = 22; 11.4%) (Figure 1).

**TABLE 1:** Clinical and demographical characteristics of the study population

	First line (N = 149)		Second line (N = 44)		All (N = 193)	
Feature	Median	Range	Median	Range	Median	Range
Age (years)	61	29 to 80	62	34 to 79	61	29 to 80
	N	%	N	%	N	%
Gender						
Males	97	65.1%	33	75.0%	130	67.4%
Females	52	34.9%	11	25.0%	63	32.6%
Performance status (WHO)						
0	106	71.1%	36	81.8%	142	73.6%
1	36	24.2%	8	18.2%	44	22.8%
2	7	4.7%	0	0.0%	7	3.6%
Site of primary tumour						
Colon	116	77.9%	33	75.0%	149	77.2%
Rectum	33	22.1%	11	25.0%	44	21.8%
Number of metastatic sites						
1	68	45.6%	25	56.8%	93	48.2%
2	50	33.6%	13	29.5%	63	32.6%
3+	31	20.8%	6	13.6%	37	19.2%
Organs involved						
Liver only	54	36.2%	14	31.8%	68	35.2%
Liver + other	72	48.3%	16	36.4%	88	45.6%
Other only	23	15.4%	14	31.8%	37	19.2%
Synchronous metastases	111	74.5%	30	68.2%	141	73.1%
ALP > 300 IU/L	42	31.8%	7	17.9%	49	28.7%
WBC > 10 × 10^9^/L	31	20.8%	5	11.6%	36	18.8%
Adjuvant chemotherapy	33	22.1%	9	20.5%	42	21.8%
Prior chemotherapy for metastatic disease^a^						
Fluoropyrimidine			41	93.2%		
Oxaliplatin	NA		20	45.5%	NA	
Irinotecan			13	29.5%		
Other			5	11.4%		

^a^ More than one drug per patient was possible.

All the 44 patients receiving chronoIFLO5 in the second‐line setting had displayed resistance to first‐line treatment, with either disease progression on first‐line chemotherapy or early relapse after completion of adjuvant chemotherapy. Additionally, 10 patients (22.7%) had also received prior adjuvant chemotherapy, and 11 patients (25%) had received prior radiotherapy. Moreover, 41 patients (93.2%) had primary tumour resection, while metastases surgery had been performed for 21 patients (47.7%). Overall, the vast majority of the patients had been exposed to folinate‐modulated fluoropyrimidines, about half to oxaliplatin, and less than a third to irinotecan (Table 1). Other uncommonly used drugs in first‐line treatment included carboplatin and mitomycin C. Primary resistance to first‐line treatment had occurred in 12 patients (27.3%). Median duration of first‐line chemotherapy was 5.5 months. Second‐line chronoIFLO5 was started after a median duration of 3.9 months after completion of first‐line chemotherapy.

### 3.2 Chemotherapy

**FIGURE 3:**
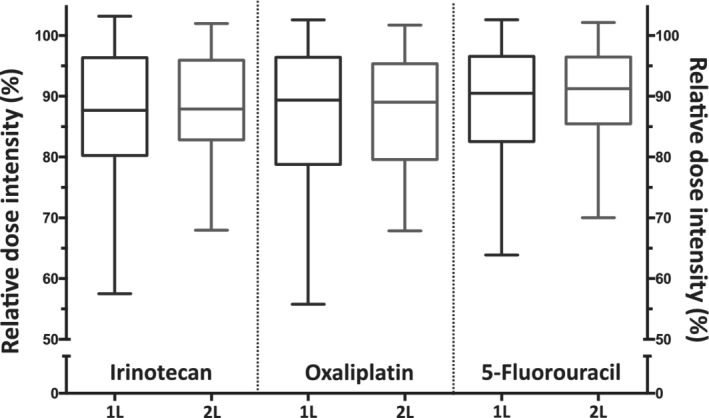
Boxplots of the relative administered dose intensities of the three main cytotoxics: irinotecan (left), oxaliplatin (centre) and 5‐fluorouracil (right), throughout the whole study, separately in first‐line (1L) and in second‐line (2L) settings

The 193 patients received a total of 1138 cycles: 905 (79.5%) as first‐line treatment and 233 (20.5%) as second‐line treatment. The median number of cycles per patient was 6 (range, 1 to 18) with frontline chronoIFLO5, and 5 (1 to 12) when used as salvage protocol. At least one dose reduction was necessary in 55% and 60% of the patients for each of the three drugs, with a similar number of patients having required at least one deferral of treatment for 5 or more days. The proportions were similar in chemotherapy naïve or previously treated patients (data not shown). Thus, median relative dose intensities of each drug in the triplet were close to 90% in both first‐ and second‐line settings (Figure 3). Only about 5% of the patients had actual relative dose intensities of less than 70% for each medication.

### 3.3 Efficacy

**FIGURE 4:**
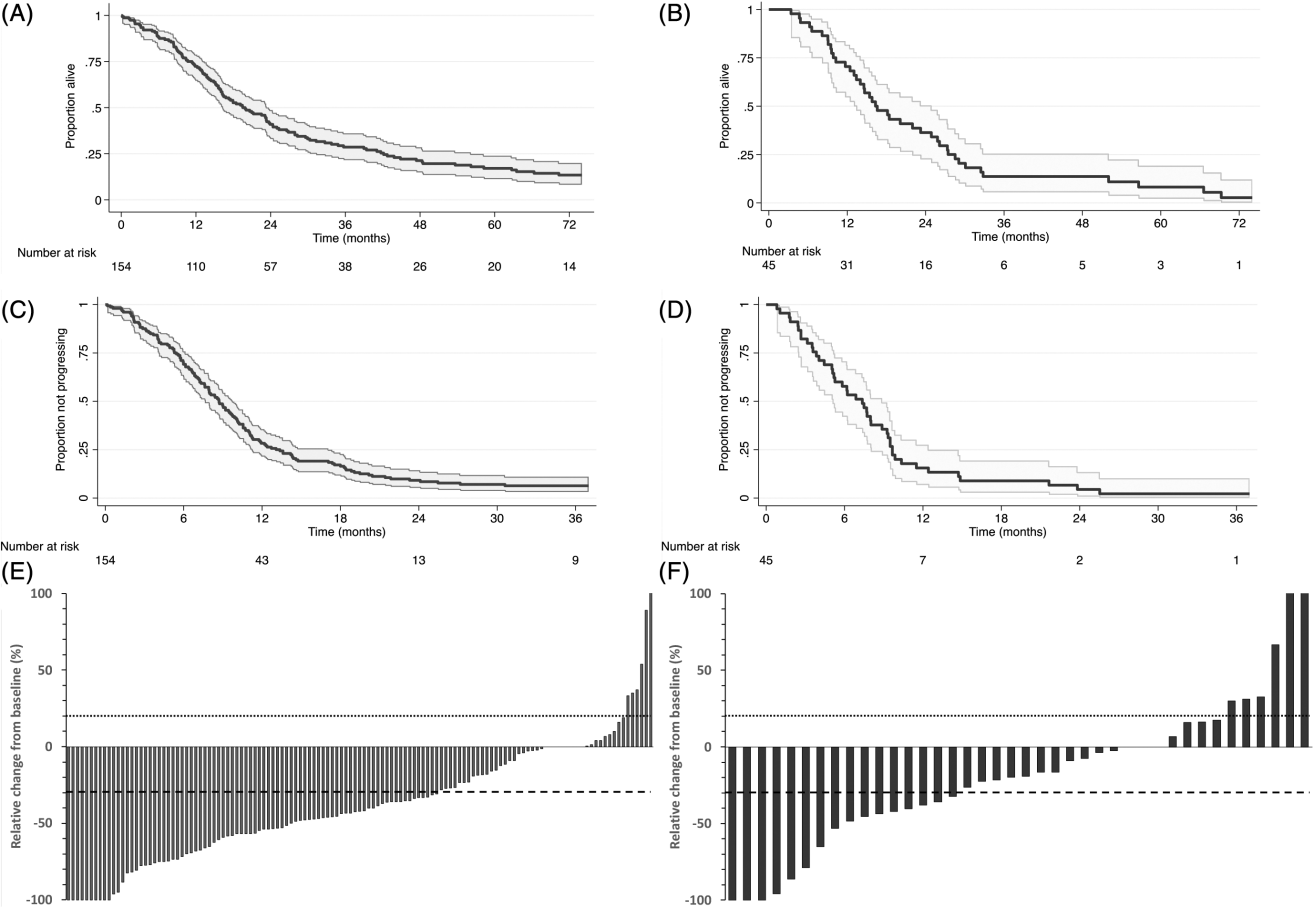
Main efficacy outcomes in patients receiving chronoIFLO5 as first‐line (left panels A, C, E) or as second‐line (right panels B, D, F) protocol. Kaplan‐Meier curves depicting overall survival (panels A, B) and progression‐free survival (panels C, D) durations, and waterfall plots showing best objective response (panels E, F)

Frontline chronoIFLO5 was associated with a median overall survival of 19.9 months [95% CI: 15.4‐24.5 months] (Figure 4A) and a median progression‐free survival of 8.7 months [7.5‐9.9] (Figure 4C). Respective figures in the second‐line setting were 16.3 months [11.8‐20.8] (Figure 4B) and 6.7 months [4.8‐8.9] (Figure 4D). Two‐ and 5‐year overall survival rates were, respectively, 41.0% [36.9‐45.1] and 16.6% [13.3‐19.9] with first‐line chronoIFLO5, and 34.9% [27.6‐42.2] and 8.4% [4.0‐12.8] when chronoIFLO5 was given as rescue second‐line protocol (Figure 4A,B).

Complete and partial radiological responses were observed in six and 80 patients, respectively, among the 138 evaluated patients on first‐line chronoIFLO5. This resulted in an objective response rate of 62.3% [54.2‐70.4] (Figure 4E). Since 39 patients also had disease stabilisation as best response, disease control rate was 90.6% [85.7‐95.5]. Second‐line chronoIFLO5 achieved one complete and 14 partial responses, and 15 disease stabilisations among 40 evaluated patients. Thus, second‐line chronoIFLO5 resulted in an objective response rate of 37.5% [22.5‐52.5] and a disease control rate of 75.0% [61.6‐88.4] (Figure 4F). Disease progression occurred in 13 patients on first line and in 10 patients in second line.

### 3.4 Safety

Overall, 58 individual serious adverse events took place in the whole trial population, with 47 being considered possibly, probably or likely caused by the administered chemotherapy. No unexpected serious adverse event occurred, and a total of 40/43 (93.0%) and 14/15 (93.3%) events resulted in an unplanned admission, in first‐ and second‐line settings, respectively.

A total of two toxic deaths occurred in first‐line treatment (1%). One patient died with Grade 5 diarrhoea, and the other one with digestive fistulisation. Protocol safety and authorisation to continue recruitment was confirmed by an Independent Data Monitoring Committee at an interim analysis after inclusion of the initial 100 patients.^14^

Out of the 149 patients receiving chronoIFLO5 as first‐line treatment, 36 (24.2%) experienced at least one severe or life‐threatening (Grade 3 or 4) toxicity. Diarrhoea (44.3%) was the most frequent clinical toxicity, whereas neutropenia was the most common haematological one, with very few febrile instances despite no G‐CSF prophylaxis (Table 2). The worst incidence of individual Grade 3‐4 toxicity per cycle was 10.6% for diarrhoea, which was the most frequent adverse event (Table 2).

**TABLE 2:** Incidence of main severe (Grades 3 and 4) toxicities per patient and per cycle, separately in first‐ and second‐line settings

	First line				Second line			
	Per patient (N = 149)		Per cycle (N = 905)		Per patient (N = 44)		Per cycle (N = 233)	
Toxicity	N	%	N	%	N	%	N	%
Haematological								
Neutropenia	21	14.6%	32	3.5%	12	27.3%	30	12.9%
Febrile neutropenia	3	2.1%	3	0.3%	0	0%	0	0%
Anaemia	6	4.2%	14	1.5%	1	2.3%	4	1.7%
Thrombocytopenia	3	2.1%	5	0.6%	1	2.3%	1	0.4%
Clinical								
Diarrhoea	66	44.3%	96	10.6%	16	36.4%	22	9.4%
Nausea	26	17.4%	38	4.2%	9	20.5%	9	3.9%
Vomiting	23	15.4%	33	3.6%	6	13.6%	9	3.9%
Asthenia	22	14.8%	29	3.2%	3	6.8%	3	1.3%
Mucositis	9	6.0%	10	1.1%	2	4.5%	2	0.9%
Sensory neuropathy	5	3.4%	5	0.6%	2	4.5%	4	1.7%
Hand‐foot syndrome	0	0%	0	0%	0	0%	0	0%

Comparable toxicity patterns were observed in the patients treated in the second‐line setting, with 10 of 44 (22.7%) having encountered at least one episode of severe toxicity. Similar to the earlier disease setting, diarrhoea and neutropenia were the Grade 3‐4 toxicities with the highest incidence per patient or per cycle (Table 2).

### 3.5 Subsequent treatments

After this triplet chronomodulated protocol, investigators had no restriction in the choice of subsequent chemotherapy drugs or regimens, if they felt further treatment was indicated.

At least one additional chemotherapy protocol was administered to 117 (78.5%) and 38 (86.4%) patients after first‐ or second‐line chronoIFLO5, respectively. Given the strict rules for protocol withdrawal for toxicity (ie, two episodes of severe toxicity despite dose reduction), a total of 67 (34.4%) patients continued the chronomodulated triplet outside the study protocol, with further dose reductions performed at the discretion of the lead clinician. Furtherly given drugs included mitomycin C, cetuximab, capecitabine, bevacizumab and carboplatin, alongside the rechallenge with either irinotecan‐ or oxaliplatin‐based regimens. Six patients (3%) received hepatic artery infusion protocols, and a total of 95 (48.7%) patients received further chronomodulated chemotherapy.

A total of 22 patients underwent metastases resection after having received chronoIFLO5, 20 (13.4%) as first‐line and 2 (4.5%) as second‐line (Figure 2) treatment. The majority of resections involved the liver (N = 20), and in two cases included also resection of the primary tumour alongside the liver. Additionally for one patient, each surgery was performed only on the pulmonary or the nodal disease. Thus, secondary hepatic surgery of initially unresectable disease was performed in 29.4% of the 68 patients with liver‐only metastases (Table 1). The 22 patients with downsized and then resected metastatic disease presented a median OS of 58.3 [37.9‐78.6] months.

## 4 DISCUSSION

In this time‐finding European prospective study, we observed rather favourable profiles of both antitumour efficacy and safety of the chronomodulated triplet combination of irinotecan, oxaliplatin and fluorouracil‐leucovorin administered every 21 days, either as frontline or as rescue treatment of metastatic colorectal adenocarcinoma. Our observations are based on unplanned descriptive analyses, exploring the outcomes separately in first‐ and second‐line.

In both settings, half of the patients received relative dose intensities of the order of at least 90% (Figure 3) and five or more cycles, assuring therefore satisfactory balance between adequate treatment intensity and duration for most patients. This converted consequently into promising objective response rate and progression‐free survival outcomes (Figure 4C‐F).

In the first‐line setting, these results compare favourably with literature reports in multicentre trials with conventional triplet regimens of the same drugs, FOLFIRINOX (irinotecan, fluorouracil, leucovorin, oxaliplatin) or FOLFOXIRI (folinic acid, 5‐fluorouracil, oxaliplatin and irinotecan),^17^, ^18^, ^19^ and even more so with less successful combination schedules.^20^, ^21^, ^22^, ^23^ Similarly favourable appears the overall tolerability of frontline chronoIFLO5 (Table 2) comparatively to literature data.^20^, ^21^, ^22^, ^23^ Moreover, although the current study was performed before molecular selection and targeted treatments became widely implemented,^24^ the overall survival observed here (Figure 4A) is of the same order of magnitude as that in the most recent studies conducted in metastatic colorectal cancer.^2^

In the second‐line setting, limited evidence currently exists for the triplet combination, beside small single‐institution studies.^11^, ^25^, ^26^ Although the various schedules were reported altogether to be feasible and active, chronoIFLO5^11^ was the only one to be tested prospectively in an international setting as salvage regimen in pretreated patients with metastatic colorectal cancer, albeit as an exploratory outcome of a tolerance‐based study. Indeed, there is concern about the risk of poor tolerance to the triplet regimen, even as frontline treatment; hence, the scarce experience reported and the preference for doublet combination.^27^ Nonetheless, the activity of second‐line doublets after failure of the other appears substantially lower than that observed in our study^28^, ^29^ (Figure 4B,D,F). However, targeted agents against EGFR or VEGF, in selected populations, meaningfully improved antitumour activity of the doublet combinations in this context.^29^, ^30^ Interestingly, monoclonal antibodies added to first‐line triplet, chronomodulated or conventional, also displayed significantly better outcomes.^10^, ^31^, ^32^ Second‐line chronomodulated triplet could further be associated with the appropriate targeted agent thus enhancing efficacy in chemorefractory disease.^13^, ^33^ In case of contraindication to the use of anti‐EGFR or anti‐VEGF agents, second‐line triplet combination could offer nonetheless arguably the highest salvage activity, especially in candidates for an onco‐surgical strategy.^34^

Although this was a time‐finding study in a cohort of unselected patients for oncosurgical strategy, the secondary resection rate in patients with liver‐only disease observed here (29.4%) compares favourably with more recent pooled evidence on downsizing systemic chemotherapy and rescue liver surgery in patients with initially unresectable colorectal liver metastases.^3^ Moreover, the median survival of the subgroup of patients undergoing secondary surgery after chronoIFLO5 approached 5 years, suggesting a long‐lasting control of the disease by this combinational chronomodulated regimen. A dedicated, prospective evaluation of this triplet is warranted to confirm our findings within an onco‐surgical strategy setting.

ChronoIFLO5 confirmed in this international setting an altogether satisfactory tolerance, especially concerning hematotoxicity rates among the lowest ones in the literature without primary G‐CSF prophylaxis (Table 2) for such highly effective triplet regimens.^17^, ^18^, ^19^, ^20^, ^21^, ^22^, ^23^ For instance, a previous trial with conventional triplet reported an incidence of Grades 3 and 4 neutropenia of 50%, with 5% instances of febrile neutropenia.^18^ Our findings here support prior evidence of a much lower neutropenia incidence with chronomodulated chemotherapy in comparison with conventional administration.^35^ Hence, chronoIFLO5 could be regarded as a possible fully‐ambulatory administration schedule of the triplet in those patients with poor tolerance to conventional delivery, as well as in other clinical scenarios where the triplet administration is an available therapeutic option, such as pancreatic ductal adenocarcinoma, or even biliary tract and gastric adenocarcinomas.^36^, ^37^, ^38^

There is evidence suggesting that circadian‐based treatment administration of anticancer drugs could be further optimised to provide supplementary benefit when individual biological clock features are taken into account.^8^ In particular, for this triplet combination, adaptation of the timing of administration of irinotecan differently in women (afternoon) and men (morning) could lead to an additional reduction in adverse events without impact on antitumour activity.^14^ Interestingly, neutropenia and appetite loss are two of the toxicities most affected by gender‐specific circadian refinement,^8^, ^14^ and also two surrogate adverse events of suboptimal chronotherapy delivery.^8^ Moreover, chronomodulated chemotherapy delivered completely at the patient's home with dedicated infusional pumps can benefit from integrative solutions of digital multidimensional remote surveillance of physiology and behaviour, whose feasibility and clinical relevance has been demonstrated expressly with this triplet regimen.^39^ This provides a novel opportunity of an adaptive closed‐loop control of cytotoxic drug administration based on patient‐generated data, unique to the fully‐ambulatory schedule.^16^

We acknowledge the limitations of our study in the current era of precision oncology, since the recruitment and treatment occurred when molecular genotype and phenotype or sideness had not yet been identified as relevant factors for treatment selection. Moreover, the time‐finding primary endpoint of the trial was toxicity based; hence, the efficacy outcomes presented here remain exploratory in nature.

Intriguingly, a lower incidence of severe diarrhoea appeared to occur with chronoIFLO5 in second‐line compared to first‐line treatment (Table 2), despite similar doses used regardless of the setting. Although more careful patient selection could have accounted for this difference, a potential adaptation of the digestive mucosa or of the intestinal microbiota^40^ to toxicity and/or of the patient's behaviour relatively to chemotherapy administration, diet and support medications could have also played a role.

In conclusion, although this international trial did not meet its primary endpoint of identifying the least toxic administration time of irinotecan, it provided prospective validation of the safety and efficacy of the chronomodulated combination of irinotecan, oxaliplatin and leucovorin‐fluorouracil administered at the patient's home. The clinical evidence reported here is the first on a multicentre basis to support the consideration of this triplet combination as an upfront and salvage regimen for patients with metastatic colorectal cancer.

## CONFLICT OF INTEREST

The authors declare no conflict of interest.

## ETHICS STATEMENT

This study was approved by the respective institutional review boards of the participating Centres and by the European Organisation for Research and Treatment of Cancer and performed in accordance with the Declaration of Helsinki.

Signed informed consent was obtained from all the patients prior to participating in the study.

## Data Availability

The database analysed in this study is securely managed by the study team and is not available as part of a public database; however, data and other items supporting the results that are minimally required to replicate the outcomes of the study will be made available upon reasonable request.
